# Attenuation of capsaicin-induced ongoing pain and secondary hyperalgesia during exposure to an immersive virtual reality environment

**DOI:** 10.1097/PR9.0000000000000790

**Published:** 2019-10-22

**Authors:** Sam W. Hughes, Hongyan Zhao, Edouard J. Auvinet, Paul H. Strutton

**Affiliations:** The Nick Davey Laboratory, Faculty of Medicine, Imperial College London, London, United Kingdom

**Keywords:** Virtual reality, Capsaicin, Endogenous analgesia, Ongoing pain, Sensitisation

## Abstract

**Introduction:**

There is growing evidence that virtual reality (VR) can be used in the treatment of chronic pain conditions. However, further research is required to better understand the analgesic mechanisms during sensitised pain states.

**Objectives:**

We examined the effects of an immersive polar VR environment on capsaicin-induced ongoing pain and secondary hyperalgesia. We also investigated whether the degree of analgesia was related to baseline conditioned pain modulation (CPM) responses.

**Methods:**

Nineteen subjects had baseline CPM and electrical pain perception (EPP) thresholds measured before the topical application of capsaicin cream. Visual analogue scale ratings were measured to track the development of an ongoing pain state, and EPP thresholds were used to measure secondary hyperalgesia. The effects of a passive polar VR environment on ongoing pain and secondary hyperalgesia were compared with sham VR (ie, 2D monitor screen) in responders to capsaicin (n = 15).

**Results:**

Virtual reality was associated with a transient reduction in ongoing pain and an increase in EPP thresholds in an area of secondary hyperalgesia. Baseline CPM measurements showed a significant correlation with VR-induced changes in secondary hyperalgesia, but not with VR-induced changes in ongoing pain perception. There was no correlation between VR-induced changes in pain perception and VR-induced changes in secondary hyperalgesia.

**Conclusion:**

Virtual reality can reduce the perception of capsaicin-induced ongoing pain and secondary hyperalgesia. We also show that CPM may provide a means by which to identify individuals likely to respond to VR therapy.

## 1. Introduction

Virtual reality (VR) interventions have shown promise as a novel distraction-based analgesic therapy for use in painful medical procedures^5,10,12,13^ and during acute pain states.^15,16,33^ There is now a growing body of evidence that suggests VR can be used in the management of chronic pain conditions^21–23,35,38,45^ and visual distraction is a key component of cognitive behavioural therapy.^24^ However, there has been limited investigation into the analgesic mechanisms of VR stimulation during sensitised pain states.

Distraction-based analgesia is a form of nonpharmacological therapy that has been shown to alter the perception of acute pain by reducing the activity within pain-related brain regions.^1,15^ The use of an immersive virtual environment has been shown to be effective at reducing the perception of pain during dental procedures that normally require local anaesthesia.^5^ The use of VR in distraction analgesia has also shown clinical utility during wound debridement associated with severe to excruciating pain in burn patients.^10,13^ Interestingly, recent advances have shown that it is possible to predict the efficacy of VR in acute experimental pain conditions by measuring the efficiency of endogenous pain inhibitory pathways using the conditioned pain modulation (CPM) paradigm.^3^

Attempts to apply immersive VR environments to chronic or ongoing pain conditions are still very much in their infancy.^23^ The majority of studies to date have typically involved measuring changes in pain perception (ie, pain scores) in groups of patients with mixed aetiologies.^21,22,45^ However, there is a distinct lack of research into whether VR can reduce altered nociceptive processing associated with the development of sensitised pain states (ie, central sensitisation^46^). The capsaicin model can be used to measure distinct chronic pain features in healthy volunteers, including spinal representations of secondary hyperalgesia and ongoing pain sensitivity.^8,30^ It provides a means by which to determine the analgesic mechanisms associated with immersion within a VR environment and may shed light on the future clinical utility of VR as a novel analgesic therapy in the treatment of chronic pain conditions.

These lines of evidence have led us to examine the analgesic mechanisms of an immersive VR environment during an experimentally induced sensitised pain state by measuring the effects on capsaicin-induced ongoing pain perception and secondary hyperalgesia in healthy volunteers. We also wanted to determine whether the efficiency of CPM measured at baseline could be used to identify individuals who were more likely to have a stronger analgesic response to VR after the onset of capsaicin-induced pain sensitivity.

## 2. Materials and methods

### 2.1. Subject screening and recruitment

All procedures were approved by the Imperial College Research Ethics Committee (18IC4435). All participants were informed of the experimental protocols and subsequently provided written consent in accordance with the principles of the Declaration of Helsinki. All subjects were recruited from Imperial College London and were initially screened to see if they met any of the exclusion criteria for pain testing (ie, pregnancy, diabetes, blood disorders, neurological conditions, immune suppression, inflammatory disease, psychiatric conditions, and taking steroid, antibiotic, or pain medicines). After initial screening, 19 healthy subjects (mean age: 24.74 ± 0.33 years; 10 females) participated in the study. According to their pain response to application of topical capsaicin cream, 15 subjects (mean age: 25.2 ± 0.47 years; 8 females) were defined as responders (ie, a maintained pain intensity rating >50 rating on a modified visual analogue scale [VAS] and a drop in pain threshold in an area of secondary hyperalgesia^8,28,42^) and 4 subjects (mean age: 23 ± 0.35 years; 2 females) were defined as nonresponders.

### 2.2. Experimental design and protocol

**Figure 1.**
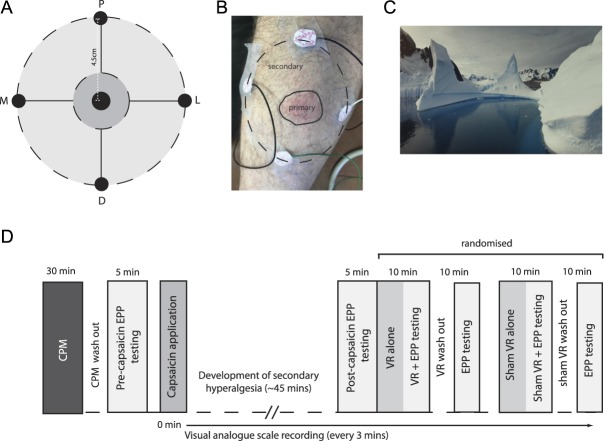
Measuring capsaicin-induced secondary hyperalgesia and experimental protocol. A) secondary hyperalgesia measurement map. Central black zone indicates position of topical application of 50 μl 1% capsaicin cream. Dark grey zone indicates the region of the neurogenic flare response (i.e. primary hyperalgesia zone). EPP thresholds are determined across proximal (P), distal (D), medial (M) and lateral (L) points in the secondary hyperalgesia zone. B) Image showing the development of a neurogenic flare response and placement of the 4 modified Ag/AgCl electrodes for measurement of secondary hyperalgesia. C) Screen capture from the Polar Obsession VR environment. D) Experimental protocol. CPM, conditioned pain modulation; EPP, electrical pain perception; VR, virtual reality.

Using a within-subject design, the effects of VR and sham VR stimulation on capsaicin-induced ongoing pain perception and secondary hyperalgesia were investigated in a randomised manner (Fig. 1). Baseline CPM responses were also examined (ie, in the absence of capsaicin).

#### 2.2.1. Conditioned pain modulation

Pressure pain thresholds (PPTs; test stimulus) were first determined by applying 3 continuous ramps of increasing intensity (0.5 kg/s) on the dominant volar forearm using a pressure algometer (WAGNER FDN 100; contact area 1 cm^2^). After a 15-minute rest, participants were instructed to immerse the non-dominant hand in ice-cold water (maintained at 8°C) up to the wrist and palm-side down for 2 minutes (ie, the cold-pressor test; conditioning stimulus). Participants were asked to rate pain perception every 10 seconds on a conventional VAS from 0 to 100 (0 = no pain; 10–30 = mild pain; 40–60 = moderate pain; 70–90 = severe pain; and 100 = worst pain imaginable). Pressure pain thresholds (ie, test stimulus) were then immediately determined by reapplying 3 continuous ramps of increasing intensity (0.5 kg/s) to the dominant forearm.^37,48^

#### 2.2.2. Baseline electrical pain perception threshold testing

After a 15-minute CPM washout period,^25^ participants were then familiarised with the electrical pain perception (EPP) threshold testing. Each transcutaneous electrical stimulus consisted of a standard, constant-current 1-ms duration square pulse using a constant current stimulator (DS7A; Digitimer, Welwyn Garden City, United Kingdom).^20^ An area on the left L5 dermatome, one-third the way along a line from the left lateral femoral epicondyle to the left lateral malleolus, was marked with a nonpermanent marker and a measurement map was drawn using four 4.5-cm spokes from the central point in proximal, distal, medial, and lateral directions. Four modified Ag/AgCl electrodes (self-adhesive, 1-cm diameter; CareFusion, Basingstoke, United Kingdom) were then positioned around each of the 4 points (Fig. 1A). Pain thresholds (mA) were then determined at each of the 4 points by increasing the current intensity in 0.5-mA steps at 1 Hz and was defined as the mean of 3 intensities logged as the point at which sensation transitioned from being a “heavy tapping” sensation (ie, no pain) to a sharp “pinprick” pain.^20^

#### 2.2.3. Capsaicin pain model

All Participants then received topical application of capsaicin cream (1% wt/wt; Pharmacierge, London, United Kingdom). Using a 1-mL syringe, 50 µL was ejected onto a 9-mm diameter clear plastic disk, which was then placed face-down in the centre of the measurement map, remaining in place for the remainder of the protocol (area of capsaicin skin contact: 64 mm^2^).^8^ The participants used a modified VAS used previously,^8^ where 0 = no sensation; 50 = pain threshold; and 100 = worst pain imaginable. After application of capsaicin cream, the participants were instructed to rate the sensation every 3 minutes for 120 minutes. The participants described the sensation initially as “tingling,” which increased in intensity over approximately 45 minutes until a distinct “stinging” or “burning” pain was perceived (ie, 50 VAS rating). Capsaicin responders were defined as participants who had established a stable pain VAS rating >50 for at least 45 minutes.^28^

#### 2.2.4. Post-capsaicin electrical pain perception testing

Previous reports show that the mean area of punctate secondary mechanical hyperalgesia after topical application of 50-µL 1% capsaicin cream is 98.9 cm^2^.^8^ Secondary hyperalgesia was then measured by testing EPP thresholds around the 4 points that covered an area of 64 cm^2^ and avoided the neurogenic flare response area (Fig. 1B).

Virtual reality headset: An Oculus Rift VR headset connected to an MSI GT83 8RF laptop (Intel Core i7-8850H 2.6-GHz processor with NVIDIA GTX 1070 SLI 8 GB graphics card) was used to display the passive virtual environment (Polar Obsession; National Geographic; Fig. 1C). Participants were seated on a couch with knee extended to 180° and the Oculus Rift motion sensor provided position and orientation data regarding the subject's head. The tracker's sensor component was mounted on the head-mounted display, and its source component was mounted on an adjustable tripod placed in front of the couch. Sham VR stimulation consisted of playing the same video on a computer monitor screen.

#### 2.2.5. Virtual reality assessment

Virtual reality or sham VR stimulation was then given in 10-minute blocks, separated by 10-minute rest periods in a randomised manner (Fig. 1D). Stimulation blocks comprised 5 minutes of VR or sham stimulation alone followed by 5 minutes of VR or sham + EPP testing. Visual analogue scale scores were recorded at the end of each stimulation block. Each 10-minute rest period comprised a 5-minute washout followed by 5 minutes of post-VR or sham stimulation EPP testing. Visual analogue scale scores were recorded at the end of each rest period.

At the end of the study protocol, each participant was given an ice pack to cool any residual burning sensation and advised to repeat if any rekindling occurred over 24 hours.

### 2.3. Statistical analysis

All data were initially entered into Microsoft Excel before being analysed for normality and statistical significance in GraphPad Prism (v8.0.1. GraphPad Software, Inc). Percentage change in CPM effect was calculated as the conditioned PPT test stimulus minus the baseline PPT test stimulus divided by the baseline PPT test stimulus.^47^ Therefore, more positive values indicated more efficient CPM. Electrical pain perception thresholds were averaged across all 4 points of the measurement map (ie, proximal, distal, medial, and lateral). Paired *t*-tests were used to analyse the changes in EPP threshold after application of capsaicin. One-way RM analysis of variance with Holm–Sidak multiple comparison post hoc analysis was used to analyse the changes in raw EPP threshold or VAS rating before, during, and after either the real or sham VR stimulation. The difference between the percentage change in VAS rating from post-capsaicin to during VR/sham was analysed by paired test. One-way RM analysis of variance with Holm–Sidak multiple comparison post hoc analysis was also used for comparing differences in the percentage change in EPP between pre- and post-capsaicin and between post-capsaicin and VR or sham VR conditions. Pearson correlation coefficient analysis was used to look for relationships between the VR-induced change in secondary hyperalgesia and CPM and the VR-induced change in pain perception and CPM. Pearson correlation analysis was also used to look for relationships between VR-induced changes in pain perception and VR-induced changes in secondary hyperalgesia. Statistical significance was set at *P* < 0.05, and all data are presented as mean ± SEM in the figures and text, where appropriate.

## 3. Results

### 3.1. Capsaicin-induced ongoing pain and electrically evoked secondary hyperalgesia

**Figure 2.**
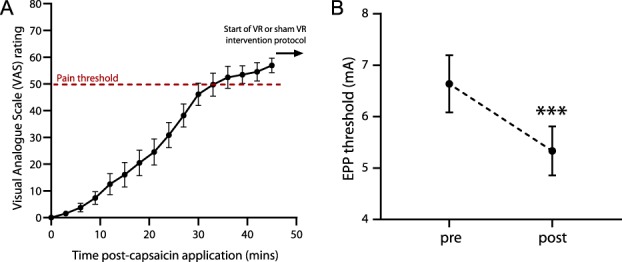
Development of capsaicin-induced ongoing pain and secondary hyperalgesia. (A) Time course for the development of pain sensation after topical application of 50-µL 1% capsaicin cream. The intensity of sensation increased until pain threshold was reached at 36 minutes (ie, dotted line; 50 VAS). (B) There was a significant drop in EPP threshold in an area of capsaicin-induced secondary hyperalgesia. Data are expressed as mean ± SEM; ***P < 0.001. n = 15. EPP, electrical pain perception; VAS, visual analogue scale.

Topical application of capsaicin results in the development of an intense burning sensation and a neurogenic flare response (ie, the primary zone; Fig. 1C). A slight tingling sensation (VAS: 1.53 ± 0.2) began to appear 3 minutes after the application of capsaicin cream (Fig. 2A). The intensity of the sensation gradually increased, and an obvious burning sensation was achieved 36 minutes after the application of capsaicin (VAS: 52.47 ± 1.07). After the development of a stable ongoing pain response (ie, *P* > 0.05 between 45 minutes post-capsaicin and 120 minutes post-capsaicin), there was a drop in EPP threshold in an area of capsaicin-induced secondary hyperalgesia (pre-EPP: 6.64 ± 0.14 mA vs post-EPP: 5.33 ± 0.12 mA; *P* < 0.001; Fig. 2B).

### 3.2. Virtual reality was associated with an attenuation of ongoing pain perception

**Figure 3.**
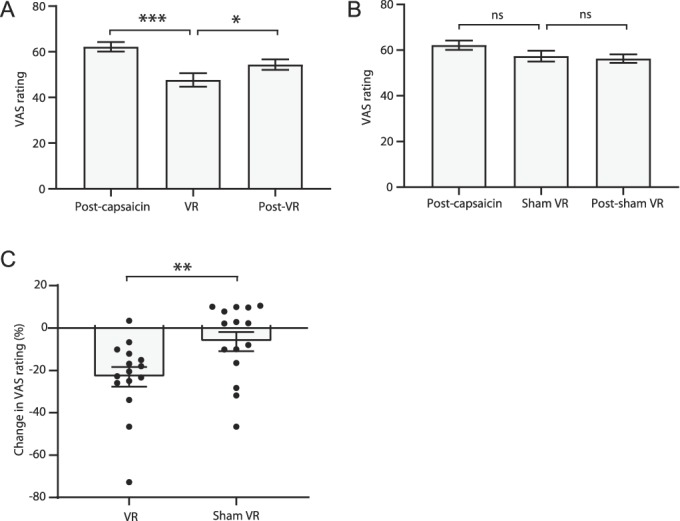
Transient changes in ongoing pain perception after VR stimulation. Changes in pain VAS ratings before, during, and after (A) real and (B) sham VR stimulation. (C) Comparison between the changes in pain rating during either real or sham VR stimulation. Data are expressed as mean ± SEM; *P < 0.05, **P < 0.01, ***P < 0.001. n = 15. VAS, visual analogue scale; VR, virtual reality.

Exposure to an immersive 3D VR environment caused a drop in VAS ratings below the defined pain threshold (post-capsaicin VAS: 62.17 ± 2.07 vs during VR VAS: 47.67 ± 2.94; *P* < 0.001; Fig. 3A), which had returned to above pain threshold (ie, > 50 VAS) when the VR headset was removed (post-VR VAS: 54.40 ± 2.29; *P* < 0.05). Sham VR was not associated with a drop in VAS rating during the stimulation (post-capsaicin VAS: 62.17 ± 2.07 vs during sham VR VAS: 57.39 ± 2.38; *P* > 0.05; Fig. 3B) and there was no difference between during and post sham VR stimulation (post-sham VR: 56.32 ± 1.87; *P* > 0.05). A paired *t* test showed a significant difference between the change in VAS rating during VR stimulation and the change in VAS rating during the sham VR stimulation (VR VAS: −23.08 ± 1.2% vs sham VAS: −6.41 ± 1.18%; *P* < 0.01; Fig. 3C).

### 3.3. Virtual reality was associated with an attenuation of secondary hyperalgesia

**Figure 4.**
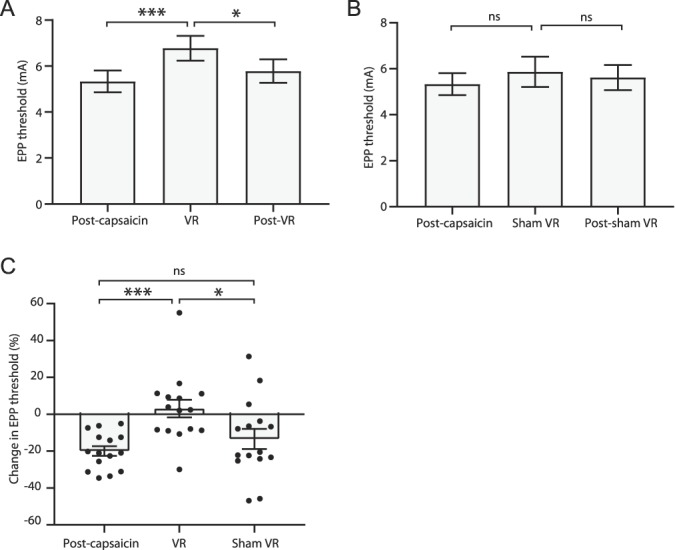
Transient changes in secondary hyperalgesia after VR stimulation. Changes in EPP threshold before, during, and after (A) real and (B) sham VR stimulation. (C) Comparison between changes in EPP threshold between real and sham VR with respect to sensitised post-capsaicin EPP thresholds. Data are expressed as mean ± SEM; *P < 0.05, ***P < 0.001. n = 15. EPP, electrical pain perception; VR, virtual reality.

There was a significant increase in EPP threshold during VR stimulation (post-capsaicin EPP threshold: 5.33 ± 0.47 mA vs during VR EPP threshold: 6.78 ± 0.54 mA; *P* < 0.001; Fig. 4A), which was reversed after removal of the VR headset (post-VR EPP threshold: 5.78 ± 0.51 mA; *P* < 0.05). Sham VR stimulation was not associated with a change in EPP threshold during (5.87 ± 0.65 mA; *P* > 0.05; Fig. 4B) or after (5.62 ± 0.55 mA; *P* > 0.05) the stimulation. Further analysis revealed there to be a significant difference in change in EPP threshold compared with post-capsaicin between VR and sham stimulation (VR: 3.05 ± 1.25% vs sham EPP: −13.37 ± 1.42%; *P* < 0.05; Fig. 4C).

### 3.4. Relationships between baseline conditioned pain modulation responses and virtual reality–induced analgesia

**Figure 5.**
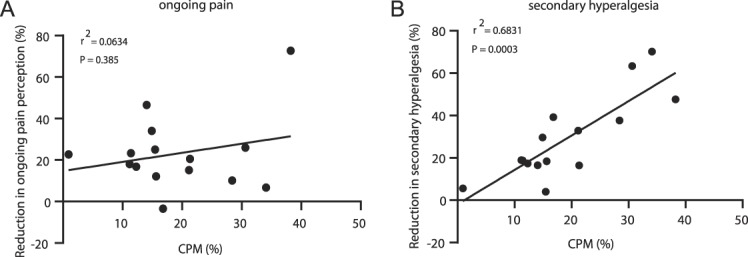
Baseline CPM correlated with VR-induced changes in secondary hyperalgesia but not VR-induced changes in pain perception. (A) No relationship between the VR-induced reduction in ongoing pain perception and levels of CPM. (B) Reduction in secondary hyperalgesia is related to baseline CPM levels. n = 15. CPM, conditioned pain modulation; VR, virtual reality.

Pressure pain thresholds were increased after the conditioning stimulus and therefore more efficient CPM is represented as a more positive value (Fig. 5). There was no correlation between VR-induced decrease in VAS and CPM (*r*^2^ = 0.063, *P* > 0.05; Fig. 5A). However, a significant correlation was found between VR-induced reduction in secondary hyperalgesia and CPM (*r*^2^ = 0.68, *P* < 0.001; Fig. 5B) in that higher levels of CPM measured at baseline (ie, in the absence of capsaicin-induced sensitivity) were associated with a greater reduction in secondary hyperalgesia. There was no relationship between VR-induced changes in pain perception and VR-induced changes in secondary hyperalgesia (*r*^2^ = 0.02; *P* = 0.6).

## 4. Discussion

We have investigated the effects of distraction using an immersive VR environment on experimentally induced ongoing pain sensitivity and secondary hyperalgesia. Using the capsaicin model of ongoing afferent drive in healthy volunteers, we show an attenuation of ongoing pain ratings and electrically evoked secondary hyperalgesia during exposure to an immersive, polar VR environment. Interestingly, we found that the magnitude of CPM measured at baseline was related to the VR-induced analgesic effect over secondary hyperalgesia but not the changes in pain ratings. These findings suggest that exposure to an immersive polar VR environment can be used to alter sensitised pain states and that CPM may be able to predict the efficacy of VR therapy in the treatment of chronic pain associated with hyperalgesia.

It has previously been speculated that VR stimulation may provide a new approach for pain management during chronic pain conditions by distracting attention away from the ongoing pain.^23,39^ Our study provides new evidence that VR stimulation can reduce capsaicin-induced ongoing pain perception. We also show novel mechanistic insight, which supports the use of VR in patients with altered nociceptive processing and central sensitisation by showing that exposure to an immersive VR environment can increase pain thresholds in an area of spinally mediated enhanced pain sensitivity (ie, secondary hyperalgesia).

In the current study, we used a passive VR design that created the impression of an immersive arctic scene during capsaicin-induced pain, which is often described as “burning.” This is in line with a previous study which demonstrated, using an immersive cold environment, that it is possible to reduce the perception of acute heat pain in healthy volunteers.^15^ It is therefore possible that the counteracting nature of cold VR environments has the ability to reduce the perception of an ongoing heat or burning pain. In this study, we found that immersion within a virtual polar world could produce robust analgesic effects in the absence of any interactive elements, which could make this approach favourable for use in patients with upper-limb mobility issues. However, it has been previously suggested that the magnitude of VR-induced analgesia is influenced by the addition of an interactive element^18,41^ as well as the quality of the visual display through the VR headset.^17^ The majority of studies using acute pain or pain tolerance paradigms have adopted interactive designs with a view to optimise the shift in attention away from the pain and into the virtual world.^5,10,12,13,15,16,33^ It is therefore possible that the analgesic effects seen in this study could be further enhanced if an interactive element is used within the VR design.

Previous research in chronic pain patients has focused on using VR involving the active navigation through fantasy landscapes and has been shown to reduce pain ratings in groups of patients with different aetiologies.^21,22^ Despite showing clinically relevant reductions in pain ratings, applying a single VR design to a mixed group of patients did not show any pain relief at all in 10% of those recruited.^22^ This could be attributed to a misalignment with the type of pain experienced by individual patients and the choice of VR environment. It is possible that specific VR environments could be more effective if aligned with the types of pain experienced, such as the use of a “snow world” in burns patients.^11^ In line with this, a case study demonstrated clinically relevant pain relief using an interactive cold environment in a treatment-resistant radiculopathy patient.^35^ Future randomised controlled trials comparing the efficacy of different types of VR design in well-defined populations of chronic pain patients may therefore lead to more targeted use of VR therapy based on the types of pain experienced.

We also show that during exposure to an immersive VR environment, it is also possible to reduce capsaicin-induced secondary hyperalgesia by measuring changes in electrically evoked pain thresholds. Previous research has shown that VR can be used to modulate acute pain thresholds in the absence of capsaicin-induced central sensitisation.^9^ To the best of our knowledge, our study is the first to show an analgesic effect over spinally mediated sensitised pain thresholds during exposure to a passive VR environment. Interestingly, we found no correlation between the VR-induced changes in ongoing pain perception and the VR-induced changes secondary hyperalgesia. This could either be explained through the different outcome measures obtained (ie, pain scores vs pain thresholds) or that VR can exert analgesic effects on pain perception and secondary hyperalgesia through 2 separate mechanisms.

Previous neuroimaging studies have shown that exposure to an immersive VR environment can activate a network of pain-related brain regions involved in top-down inhibitory control^14,15^ and that there is an increase in functional connectivity between the medial prefrontal cortex and spinally projecting centres in the midbrain and brainstem during distraction-based analgesia.^4,29,36,40^ It is therefore possible that VR can engage similar top-down analgesic pathways involved in the descending modulation of spinal cord nociceptive processing in a manner akin to that seen during noninvasive brain stimulation.^19,20,31^ The analgesic effects on capsaicin-induced ongoing pain ratings could be due to activation of a corticocortical analgesic pathway associated with emotion and memories as well as auditory or visual stimuli, which is distinct from the top-down activation of the descending pain modulatory network.^7,43^

It has been previously shown that measuring CPM at baseline can be used to identify individuals likely to benefit from VR during an acute pain tolerance test.^3^ This study extends these findings to show that CPM can be also be used to identify those likely to show a greater reduction in experimentally induced secondary hyperalgesia. It is becoming increasingly clear that CPM is not just dependent on activation of a spinobulbospinal loop mechanism first proposed in rodents,^26,27^ but is also open to interaction with psychological and cognitive factors.^6,34^ It is therefore possible that the association observed in this study is due to distraction playing a role in both the CPM effects as well as the VR-induced changes in secondary hyperalgesia. This is supported through recent neuroimaging studies, which have shown that both CPM and VR are associated with activation of the anterior cingulate cortex,^15,32^ an area of the brain closely linked with distraction analgesia that has been shown to be involved in top-down pain control.^2,14–16^ Conditioned pain modulation has been previously shown to predict the response to duloxetine in patients with diabetic neuropathy, by mimicking the action of descending monoaminergic inhibitory pathways.^48^ The association seen between CPM and VR-induced changes in secondary hyperalgesia suggests that VR may exert a top-down influence on similar spinally projecting descending control pathways.^43,44^ Another possible explanation for the observed relationship between CPM and VR-induced changes in secondary hyperalgesia is that they are both measuring changes in pain threshold. However, because we were testing 2 separate modalities in these paradigms (ie, PPT and EPP), it is more likely that the CPM response and the VR-induced changes in secondary hyperalgesia share similar top-down mechanisms. To confirm this, further psychophysical and neuroimaging research is required to show whether exposure to VR can modulate the CPM response and activate common pain-related brain and brainstem regions.

This proof-of-concept study is the first to show evidence that VR stimulation can modulate altered nociceptive processing associated with the development of chronic pain states. However, there are a few limitations with our study that should be addressed in larger randomised controlled trials. We used a within-subject design to test the effects of short periods of VR or sham VR stimulation on established pain sensitivity. We found the effects to be transient and dependent on the presence of the VR environment. Future studies adopting a crossover design with participants randomly assigned to either real or sham VR conditions will allow longer stimulation times and measurement of the time course of the analgesic effects after cessation of the VR environment. It would also be of interest to investigate the effects of VR on other measures of central sensitisation such as dynamic mechanical allodynia or whether it is possible to reduce areas of punctate mechanical secondary hyperalgesia.

In summary, the study presented here demonstrates the first evidence that a passive VR design can reduce both ongoing pain perception and secondary hyperalgesia during an experimentally induced sensitised pain state. We show that exposure to a cold immersive VR environment may be a promising new analgesic therapy for use in chronic pain patients by providing a novel nonpharmacological approach to alleviate the severity of ongoing pain and associated spinal cord excitability. It may also be possible to use CPM to guide future phenotype-stratified trials with VR in the treatment of chronic pain.

## Disclosures

The authors report no conflicts of interest.
